# Migration history, first episode psychosis and child abuse: Results from the EU-GEI study

**DOI:** 10.1192/j.eurpsy.2021.860

**Published:** 2021-08-13

**Authors:** I. Tarricone, J. Lal, G. D’Andrea, R. Muratori, C. Morgan, D. Berardi, R. Murray, M. Di Forti

**Affiliations:** 1 Department Of Medical And Surgical Sciences, Bologna University, Bologna, Italy; 2 Dibinem, Bologna University, Bologna, Italy; 3 Department Of Mental Health And Pathological Addictions A, Bologna Local Health Authority Lo, Bologna, Italy; 4 Institute Of Psychiatry, Psychology & Neuroscience | Health Service And Population Research, King’s College London, London, United Kingdom; 5 Institute Of Psychiatry, Psychology & Neuroscience, King’s College London, London, United Kingdom

**Keywords:** migration history, First episode psychosis, child abuse

## Abstract

**Introduction:**

Child abuse is associated with a wide range of mental disease including psychotic disorders. Few studies have investigated the role of child abuse in contributing to increase the risk of psychosis in migrant population.

**Objectives:**

To explore the risk of first episode psychosis (FEP) in migrants and natives for each type of trauma i.e. physical abuse (P.A.), sexual abuse (S.A.), emotional abuse (E.A.), physical neglect (P.N.) and emotional neglect (E.N.).

**Methods:**

Within a large case- control incidence sample of FEP from the EU-GEI study (The EUropean Network of National Schizophrenia Networks Studying Gene–Environment Interactions) we evalued the assocition of childhood trauma with FEP in migrants and natives. Associations were adjusted for age, gender, social status, level of education, family history of psychosis and cannabis use. Trauma was assessed through Childhood Trauma Questionnaire (CTQ).

**Results:**

CTQ mean score was higher in FEP migrants (45.4, sd 15.6) than in FEP natives (41.7, sd 13.9) (p = 0.002). In natives every type of child abuse was associated with FEP. In migrants P.A., S.A., P.N. were associated with FEP. We found a dose – dependent relationship between trauma and FEP.

**Conclusions:**

Child abuse is common in individuals with psychosis. FEP migrants are more exposed to childhood trauma. Clinicians should routinely assess patients for childhood trauma. When treating a FEP migrant patient, clinicians must be aware of an underlying traumatic childhood adversity more than of a traumatic migration history.

